# Cognitive Symptoms Are Not Associated with Cognitive Performance in Post-Acute mTBI

**DOI:** 10.1093/arclin/acae060

**Published:** 2024-08-08

**Authors:** Arielle M Levy, Michael M Saling, Jacqueline F I Anderson

**Affiliations:** Melbourne School of Psychological Sciences, The University of Melbourne, Melbourne, Victoria, 3010, Australia; Melbourne School of Psychological Sciences, The University of Melbourne, Melbourne, Victoria, 3010, Australia; Melbourne School of Psychological Sciences, The University of Melbourne, Melbourne, Victoria, 3010, Australia; Psychology Department, The Alfred hospital, Commercial Rd, Melbourne, Victoria, 3004, Australia

**Keywords:** Head injury, Traumatic brain injury, Attention, Learning and memory, Executive functions, Assessment, Rehabilitation

## Abstract

**Objective:**

Subjective cognitive symptoms are commonly reported after mild traumatic brain injury (mTBI) but are often not associated with objective cognitive performance. This may be due to limitations in traditional cognitive performance measures, which may not be sensitive to subtle variations in cognition in post-acute mTBI. This study explored associations between objective and subjective cognition using computer-based tasks of increasing cognitive load, proposed to be more sensitive to subtle differences in performance.

**Method:**

Individuals with mTBI (n = 68) and trauma controls (n = 40) were prospectively recruited and assessed approximately 8 weeks post-injury. Participants completed measures of subjective symptom reporting, objective cognitive performance (including two computer-based tasks of increasing cognitive load), and psychological distress.

**Results:**

There were no significant associations between subjective and objective cognition reporting in the mTBI group, both in bivariate correlations (|*r*| = 0.01–0.20, *p* > .05) and when controlling for psychological distress (|*r*| = 0.00–0.17, *p* > .05). A similar pattern of results was observed in trauma controls, suggesting that the limited relationships between objective and subjective cognition in mTBI may not be specific to this population.

**Conclusions:**

Despite employing measures of cognitive performance proposed to be more sensitive than traditional tasks, no significant relationships were observed between objective and subjective cognition in post-acute mTBI, and estimated effect sizes were small to negligible. This provides further evidence that at a group level 8 weeks after mTBI subjective cognitive symptoms primarily reflect factors aside from objective cognition.

## INTRODUCTION

Mild traumatic brain injury (mTBI) is common, with an estimated 42 million individuals affected worldwide each year and rates seemingly increasing (Cancelliere et al., 2017; Cassidy et al., 2004; Gardner & Yaffe, 2015). Many individuals experience prolonged recovery following mTBI, with recent research finding that over 50% of patients continue to subjectively report “post-concussion” symptoms at 1 year post-injury (Machamer et al., 2022). The prevalence of mTBI, and high incidence of poor recovery, makes it an important area for continued research.

One of the most common types of post-concussion symptoms reported after mTBI is cognitive symptoms, which refers to subjective reports of decreased cognitive functioning. These subjective cognitive deficits are typically reported in the domains of processing speed, attention, memory, and executive functioning (Clarke et al., 2012). As with other post-concussion symptoms reported after mTBI, cognitive symptoms can persist for a year or longer following injury (Machamer et al., 2022; Theadom et al., 2018). Additionally, cognitive symptoms are likely to be of specific importance in mTBI given their relative specificity in mTBI compared with varied control groups (Levy et al., 2022) and their association with return to work (Phillips et al., 2017; Theadom et al., 2017).

In addition to subjectively-reported symptoms, objective cognitive impairments are common in the initial weeks and months following mTBI (Carroll et al., 2004b; Carroll et al., 2014). These objective impairments are typically observed in the same cognitive domains as subjectively-reported symptoms: processing speed, attention, memory, and executive functioning (Rabinowitz & Levin, 2014). Understanding whether subtle cognitive deficits underlie the subjective cognitive symptoms frequently reported after mTBI may lead to better management strategies for individuals with persistent symptoms (Levy et al., 2022; Ngwenya et al., 2018). To date, studies exploring the relationship between objective and subjective cognition in post-acute mTBI have produced mixed results. Although some studies have found a relationship between cognitive performance and cognitive symptoms (Chamelian & Feinstein, 2006; Clarke et al., 2012; Ngwenya et al., 2018), other studies have not found a relationship between the two factors (Anderson, 2021; Stulemeijer et al., 2007).

One reason for these mixed findings may be due to the use of insufficiently sensitive cognitive performance measures when exploring this relationship. Specifically, it has been suggested that the traditional neuropsychological measures commonly employed in mTBI-related research may not be sensitive enough to detect subtle cognitive deficits that may be present in the post-acute period (Bigler, 2013; Sparadeo et al., 2018). Two types of tasks that have been proposed to potentially be more sensitive to subtle deficits are computer-based tasks and tasks of increasing cognitive load (Bigler, 2013; Dean & Sterr, 2013). Computer based tasks have reduced measurement error relative to traditional paper-and-pencil based tasks and can measure response times in milliseconds rather than seconds, theoretically allowing for the detection of more subtle variations in performance (Bigler, 2013; Rabinowitz et al., 2015). Similarly, tasks of increasing cognitive load are considered more likely to be sufficiently cognitively demanding to detect subtle cognitive deficits (Dean & Sterr, 2013; Ozen & Fernandes, 2012). Due to their proposed sensitivity, computer-based tasks of increasing cognitive load may be useful when exploring the relationship between objective and subjective cognition, particularly during the post-acute period when cognitive deficits would be expected to be less prominent than during the acute period (Karr et al., 2014). Given that cognitive symptoms often remain elevated throughout the post-acute period (Levy et al., 2023), research is needed to understand whether subtle cognitive deficits may underly these ongoing symptoms.

Another key component in understanding the relationship between objective and subjective cognition in mTBI involves controlling for general trauma-related factors which might impact this relationship in mTBI. A recent study found a significant relationship between a measure of cognitive performance and cognitive symptoms in healthy controls but not in a group of individuals with mTBI, suggesting that the experience of mTBI may change the relationship between subjective and objective cognition (Anderson, 2021). However, there are many trauma-related differences between mTBI and healthy control samples aside from the experience of mTBI itself, which might impact the relationship between cognition and complaint. For this reason, it is beneficial to control for these potentially confounding variables by employing a comparison group of individuals with general trauma. A prior study that did employ trauma controls found significant linear associations between objective and subjective cognition in mTBI but not in healthy or trauma controls (Clarke et al., 2012). These findings directly contradict those of the more recent study (Anderson, 2021), indicating that the relationship between objective and subjective cognition in mTBI warrants further investigation.

In order to comprehensively examine the relationship between objective and subjective cognition in mTBI, it is necessary to also explore this relationship using computer-based measures of increasing cognitive load, with the inclusion of a sample of well-matched trauma controls. The primary aim of this study was to understand the relationship between cognitive performance and cognitive symptom reporting in post-acute mTBI, using potentially more sensitive measures of cognitive performance and a well-matched group of trauma controls. It is hypothesized that there will be a small but significant relationship between objective and subjective cognition in mTBI when using these potentially more sensitive measures of cognitive performance.

## METHODS

### Participants

A total of 68 mTBI and 40 trauma control (TC) participants were included in the current study. Participants were recruited from inpatient trauma wards at The Alfred hospital and Royal Melbourne Hospital in Melbourne, Australia. Participant eligibility and group membership were assessed using information from hospital records and patient interview. Eligible participants were assigned to either the mTBI or the TC group based on the nature of their injury. Individuals in the mTBI group were those who had sustained an mTBI as per the World Health Organization definition (Carroll et al., 2004a). Individuals were assigned to the TC group if they had sustained general traumatic injuries but had not sustained a head injury or head strike. General exclusion criteria for both groups was: age < 18 or > 60 years; recent premorbid mood/anxiety disorder (i.e., active symptoms or treatment within the 12 months prior to injury); history of severe psychological condition; previous moderate–severe head injury; previous neurological history; current or prior intravenous drug use; current or prior heavy alcohol use (average of >6 standard drinks per day); or lack of English proficiency. An additional exclusion criterion for the trauma control group was a history of previous mTBI or concussion. More detailed information on participant recruitment and eligibility can be found in a previous publication (Anderson & Fitzgerald, 2018). Approval for this study was granted by the Human Research Ethics Committees at The Alfred hospital and Royal Melbourne Hospital. Recruitment for participants included in this study occurred between December 2016 and January 2020. This study employs the same participant sample as a previous publication (Levy et al., 2023); a flow chart of participant recruitment is provided therein.

### Measures

#### Symptom reporting

##### Cognitive symptoms

Subjective cognitive symptoms were measured using the Cognitive Complaint After Mild Closed Head Injury scale (CCAMCHI; Anderson, 2021; Clarke et al., 2012). The CCAMCHI is a 30-item scale that measures subjective change in cognitive abilities in the domains of processing speed, memory, attention, and executive function (Anderson, 2021). This scale incorporates more cognitive items than post-concussion scales which are traditionally used to assess cognitive symptoms in mTBI, and thus is likely to provide a more reliable estimate of subjective cognition (Anderson, 2021). The CCAMCHI scale has been used previously in mTBI research (Anderson, 2021; Levy et al., 2023), and has excellent reliability in this population (Levy et al., 2023).

##### Post-concussion symptoms

General post-concussion symptoms were evaluated using the Rivermead Post-Concussion Symptoms Questionnaire (RPQ), which is widely used in mTBI research (Cassidy et al., 2014; Levy et al., 2022). On this scale, participants rate the degree to which they currently experience symptoms relative to pre-injury, for 16 common cognitive, affective, and somatic symptoms (King et al., 1995). This scale was included to enable comparison to previous research.

#### Cognitive performance – Increasing cognitive load tasks

##### n-back

The n-back task is a measure of working memory that has been used previously in mTBI research (Dean & Sterr, 2013; Wylie et al., 2015). During the task, letters were presented one at a time on screen, and individuals were required to respond via a key press when a given condition was met. For the 0-back level, individuals responded when the letter “x” was presented. For the 1-, 2-, and 3-back conditions, individuals were required to respond when the present letter matched the letter presented 1, 2, or 3 letters previously. Conditions were presented in increasing level of difficulty, beginning with the 0-back and concluding with the 3-back. The number of target trials for the 0-back, 1-back, 2-back, and 3-back conditions were 23, 24, 24, and 11, respectively. Trials that were outliers on response time (z > 3.29, Tabachnick & Fidell, 2013) were removed for each participant (Mathias et al., 2004). The total number of errors (omission + commission) was calculated for each participant at each level.

##### Increasing Distractors Paradigm

The Increasing Distractors Paradigm (IDP) is a reaction time task that was developed to detect subtle cognitive changes following brain injury has been employed with mTBI participants (Anderson et al., 2008; Anderson & Cockle, 2021). During this task, participants were presented with 1, 2, 4, 6, or 8 stimulus circles on screen (levels 1–5, respectively), each corresponding to a key on the keyboard. For each trial, a warning tone was played, followed by an interstimulus interval of 1–4 s, and then a random stimulus circle becoming blue. The participant was required to respond as quickly as possible to the blue circle (target) by pressing the corresponding key on the keyboard. Conditions were presented in increasing level of difficulty for each participant. Prior to the first level, the participant completed two practice conditions, and a baseline condition in which all eight stimulus circles were present, but the order of target presentation was not random and was known to the participant. A visual representation of this task is presented in a previous publication (Anderson et al., 2008). Outlier trials were removed for each participant (Mathias et al., 2004), and responses times were calculated for each participant at each task level.

#### Cognitive performance – Traditional measures

The traditional cognitive performance measures included in this study evaluate performance in the cognitive domains most commonly impacted by mTBI: processing speed, attention, memory, and executive function (Rabinowitz & Levin, 2014). These measures were included to enable comparisons to previous research. Each of these cognitive measures is used regularly in mTBI research and has acceptable psychometric properties (Sherman et al., 2022; Wilde et al., 2010). Further, each measure is included as part of the National Institute of Neurological Disorders and Stroke (NINDS) list of common data elements recommended for the assessment of mTBI (NINDS, n.d.; Wilde et al., 2010). To provide an indication of the overall level of cognitive recovery of each group, standardized scores were computed for each measure using available normative data (see Table 2 footnote).

##### Symbol Digit Modality Test

The written version of the Symbol Digit Modality Test (SDMT) was employed as a measure of processing speed (Smith, 1982). The SDMT is a speeded task that requires participants to fill in numbers that correspond to rows of symbols using a key that presents the symbol-digit pairs. The total score on this version of the task was the number of correct items completed in 120 s.

##### Digit Span

The Digit Span subtest from the Wechsler Adult Intelligence Scale, Fourth Edition was included as a measure of attention and working memory (Wechsler, 2008). This task requires individuals to repeat verbally presented strings of numbers in presentation order, reverse order, and ascending order. Total raw scores were used, rather than age-scaled scores, in order to maintain consistency with the other cognitive performance measures for which age-scaled scores were not available.

##### Rey Auditory Verbal Learning Test

The Rey Auditory Verbal Learning Test (RAVLT) is a measure of learning and memory (Schmidt, 1996; Wilde et al., 2010). On this task, individuals are read a list of 15 words (List A) and required to recall as many words as possible from the list over consecutive trials. The five learning trials are followed by the presentation and recall of a distractor list (List B), immediate and delayed recall conditions (List A), and a delayed recognition trial (List A). The total score on this task was the sum of all items recalled across the five learning trials (A1-A5).

##### Trail Making Test-Part B

The Trail Making Test-Part B (TMT-B) is a measure of executive functioning (Reitan, 1992). This task requires participants to draw lines to connect circles containing alphanumeric characters in alternating ascending order (i.e., 1-A-2-B. . .). The total score was the time taken to complete Part B of the task.

##### Controlled Oral Word Association Test

The Controlled Oral Word Association Test (COWAT) was employed as an additional measure of executive functioning (Benton et al., 1994). On this task, individuals are given 60 s (per letter) to list as many words as they can think of that begin with the given letter. The total score was the number of words produced across the three components of the task, using the letters F, A, and S.

#### Psychological distress

A psychological distress index was incorporated in the current study given that psychological distress can impact both objective and subjective cognition (Chamelian & Feinstein, 2006; Levy et al., 2023; Ngwenya et al., 2018; Schneider et al., 2022). For this reason, psychological distress is considered a key variable to control for when examining relationships between objective and subjective cognition (Burmester et al., 2016; Hromas et al., 2021).

The psychological distress index was created by combining scores on the Beck Anxiety Inventory (BAI; Beck et al., 1988), the Inventory of Depressive Symptomatology (IDS; Rush et al., 1996), and the PTSD Checklist for DSM-5 (PCL-5; Weathers et al., 2013). Each of these measures have acceptable psychometric properties (Beck et al., 1988; Blevins et al., 2015; Rush et al., 1996) and have previously been employed in mTBI (Levin et al., 2021; Stillman et al., 2020; Suhr & Gunstad, 2002). To create the psychological distress index, total scores on the PCL-5 were adjusted such that all three measures were based on a four-point rating scale, then total scores were summed across the three measures. Possible scores on this index ranged from 0 to 207, with higher scores representing greater psychological distress. Becauseinterpretation of this idiosyncratically-created index is not intuitive, average scores for each of the three measures were compared to recommended clinical thresholds (Bardhoshi et al., 2016; Blevins et al., 2015; Rush et al., 1996).

#### Estimated premorbid intelligence

The Weschler Test of Adult Reading (WTAR) was employed as a measure of estimated premorbid intellectual functioning (Wechsler, 2001). This task provides stable and reliable estimates of premorbid intelligence in individuals with mTBI (Mathias et al., 2007; Steward et al., 2017).

#### Performance validity

The Digit Span task and the RAVLT, in addition to their use as measures of cognitive performance, were also used as embedded measures of performance validity. Poor performance validity was defined as a Digit Span age-corrected standard score (ACSS) ≤5 or a RAVLT recognition score ≤ 9, thresholds which maximize sensitivity and specificity (Babikian et al., 2006; Babikian & Boone, 2007; Boone et al., 2022).

### Procedure

Participants were assessed in-person at The Alfred hospital approximately 6–10 weeks after injury. Measures were completed in the following order: SDMT, n-back, WTAR, IDP, RAVLT, DS, TMT-B, COWAT, RPQ, CCAMCHI, IDS, BAI, and PCL-5.

Individuals who scored below the cutoff on either performance validity index were excluded from the current study (n = 1 mTBI; n = 3 TCs). This resulted in the final sample of 68 mTBI participants and 40 TC participants.

### Statistical analysis

Missing data is reported in table footnotes where relevant, and was not present in any variable at a frequency greater than 5%, raising minimal concerns about potential bias (Tabachnick & Fidell, 2013). All statistical assumptions were evaluated prior to analyses. There was no more than one outlier (z > 3.29; Tabachnick & Fidell, 2013) in each group for any variable. When outliers were present, analyses were run with and without the outlier(s), and any changes to the pattern of results are reported (Aguinis et al., 2013). Welch’s two sample t-tests and Chi-squared tests were employed to compare groups on demographic, injury, and other background variables. The exception was the IDP data, which was compared using individual ANCOVAs at each task level, to control for baseline task performance. Spearman correlations were employed to assess the relationships between symptom and performance measures, given that responses on several included variables were not normally distributed (CCAMCHI, TMT-B, n-back, IDP, and psychological distress; Field, 2012). Due to the large number of correlations, the Benjamini-Hochberg method was employed to control the overall false discovery rate for each correlation table using *d* = 0.05 (Benjamini & Hochberg, 1995; Glickman et al., 2014). Effect sizes were considered alongside tests of statistical significance, as recommended in the literature (Fritz et al., 2012; Lakens, 2013; Sullivan & Feinn, 2012). Interpretation of effect sizes was based on recommended benchmarks (Cohen, 1988; Ellis, 2010). Statistical analysis was conducted using R version 4.2.1 (R Core Team, 2022), using the following packages: tidyverse (Wickham et al., 2019), finalfit (Harrison et al., 2020), rstatix (Kassambara, 2021), and psych (Revelle, 2022).

## RESULTS

The study sample consisted of 68 mTBI participants (age = 36.79, SD = 14.81; 22.1% female) and 40 TC participants (age = 37.05, SD = 12.78; 17.5% female). Demographic and injury data for each group is presented in Table 1.

**Table 1:** Demographic and injury variables for mTBI and TC groups

	mTBI (n = 68)	TC (n = 40)	*p*-value
Age, mean (SD), range	36.79 (14.81), 18–60	37.05 (12.78), 18–59	0.925
Sex	M: 53, F: 15	M: 33, F: 7	0.570
Years of education	13.34 (2.35)	13.35 (2.68)	0.982
Predicted FSIQ	105.90 (9.23)	106.03 (9.46)	0.946
Current work status	FT: 33, PT: 11, Not working: 24	FT: 20, PT: 7, Not working: 13	0.954
Days since injury, mean (SD), range	60.40 (10.39), 37–84	56.65 (12.02), 39–88	0.105
Mechanism of injury, n (%)			0.692^a^
Motor vehicle accident	10 (14.7%)	9 (22.5%)	
Motorbike accident	10 (14.7%)	9 (22.5%)	
Cycling accident	22 (32.4%)	9 (22.5%)	
Fall	13 (19.1%)	6 (15.0%)	
Sports	4 (5.9%)	3 (7.5%)	
Other	9 (13.2%)	4 (10.0%)	
Glasgow Coma Scale score, n (%)^b^			-
13	5 (7.4%)		
14	14 (20.6%)		
15	48 (70.6%)		
Loss of consciousness, n (%)^c^			-
None	10 (14.7%)		
<1 min	27 (39.7%)		
1–5 mins	20 (29.4%)		
5–10 mins	5 (7.4%)		
10–20 mins	1 (1.5%)		
20–30 mins	4 (5.9%)		
Post-traumatic amnesia, n (%)			-
<1 min	18 (26.5%)		
1–5 mins	19 (27.9%)		
5–60 mins	12 (17.6%)		
1–6 hrs	5 (7.4%)		
6–18 hrs	8 (11.8%)		
18–24 hrs	6 (8.8%)		

*Note.* FSIQ = full scale IQ; FT = full-time; mTBI = mild traumatic brain injury; PT = part-time; TC = trauma control; WTAR = Wechsler Test of Adult Reading.

^a^Based on Chi-squared test with simulated *p*-value (2000 replicates).

^b^Glasgow Coma Scale score was unknown for one mTBI participant.

^c^Loss of consciousness data was unavailable for one mTBI participant.

Groups were well-matched on all demographic and injury variables. Table 2 presents summary data for background study variables, including subjective symptoms, cognitive performance, and psychological distress variables.

**Table 2:** Symptom, cognitive performance, and psychological distress data for each group

	Raw Scores			Standardized Scores^a^	
	mTBI (n = 68)	TC (n = 40)	*p*-value	mTBI (n = 68)	TC (n = 40)
Cognitive symptoms (CCAMCHI)	94.59 (7.89)	89.73 (6.74)	**0.001**		
Postconcussion symptoms (RPQ)	12.19 (10.68)	9.63 (9.75)	0.206		
Cognitive performance					
n-back (Errors)					
0-back	1.84 (1.82)	1.50 (1.90)	0.367		
1-back	8.31 (5.76)	6.35 (5.77)	0.092		
2-back	15.90 (4.89)	15.50 (4.64)	0.729		
3-back	10.60 (2.12)	10.60 (1.80)	0.933		
IDP (RTs)					
Level 1	334.40 (89.12)	334.02 (121.57)	0.848		
Level 2	376.42 (51.33)	376.28 (71.14)	0.770		
Level 3	550.02 (127.57)	541.69 (119.96)	0.881		
Level 4	631.69 (142.31)	604.86 (122.01)	0.377		
Level 5	702.19 (148.29)	678.17 (116.21)	0.452		
Traditional measures					
SDMT	67.81 (12.15)	66.65 (13.10)	0.650	−0.16	−0.36
Digit Span	28.91 (4.91)	29.50 (4.72)	0.539	0.21	0.30
RAVLT	53.56 (8.56)	53.05 (9.57)	0.782	0.14	0.05
TMT-Part B	58.56 (17.32)	59.83 (23.80)	0.770	0.20	0.20
COWAT	36.94 (10.18)	39.78 (9.56)	0.150	−0.13	−0.06
Psychological distress index^a^	27.46 (21.19)	21.98 (15.09)	0.128^b^		

*Note.* Baseline IDP performance was included as a covariate on comparisons for each level of the IDP. CCAMCHI = Cognitive Complaint After Mild Closed Head Injury; COWAT = Controlled Oral Word Association Test; FSIQ = full scale IQ; FT = full-time; IDP = Increasing Distractors Paradigm; mTBI = mild traumatic brain injury; PT = part-time; RAVLT = Rey Auditory Verbal Learning Test; RPQ = Rivermead Post-Concussion Symptoms Questionnaire; RT = reaction time; SDMT = Symbol Digit Modalities Test; TC = trauma control; TMT-Part B = Trail Making Test-Part B.

^a^Based on available normative data, Digit Span and RAVLT scores are age-standardized, TMT-Part B and COWAT scores are age- and education-standardized, and SDMT scores are age-, education-, and sex-standardized (Kiely et al., 2014; Sherman et al., 2022; Strauss et al., 2006; Tombaugh, 2004; Wechsler, 2008).

^b^Psychological distress data was missing for one mTBI participant and two TC participants.

^c^When a single univariate TC outlier was removed, the difference between groups was significant (TC mean [SD] = 20.48 [12.08], *p* = 0.035).

The mTBI group reported significantly greater cognitive symptoms than the TC group, although there were no group differences in overall post-concussion symptoms. Additionally, there were no between-group differences in performance on any of the cognitive measures. Of note, the 0-back level of the n-back showed a potential ceiling effect of performance in both groups, although the 3-back level showed a potential floor effect of performance, with low variance at both levels. Standardized scores computed for each of the traditional measures suggested that at a group level objective cognitive performance appears to have largely recovered by the time of assessment (Table 2). Psychological distress did not significantly differ between groups when the full samples were compared; however, when a single TC outlier was removed the difference between groups became significant, with greater psychological distress in the mTBI group (see Table 2 footnote). Psychological distress for both groups fell within the normal range when compared to recommended clinical thresholds (average IDS score = 12.7 for mTBIs, 10.8 for TCs [clinical cutoff ≥18; Rush et al., 1996]; average BAI score = 6.2 for mTBIs, 5.5 for TCs [cutoffs of 7+ recommended; Bardhoshi et al., 2016]; average PCL-5 score = 11.6 for mTBIs, 7.4 for TCs [cutoffs of 28+ recommended; Blevins et al., 2015]).

### Relationships between cognitive performance and symptom reporting

Bivariate correlations between cognitive performance and cognitive symptoms are provided in Table 3. Bivariate correlations between cognitive performance and general post-concussion symptoms are also reported, to enable comparison with previous research.

**Table 3:** Bivariate correlations between cognitive performance and cognitive and post-concussion symptom reporting in each group

	mTBI (n = 68)		TC (n = 40)	
	CCAMCHI	RPQ	CCAMCHI	RPQ
n-back				
0-back	0.20 [−0.04, 0.42]	0.21 [−0.03, 0.42]	0.00 [−0.31, 0.32]	−0.11 [−0.41, 0.21]
1-back	0.13 [−0.11, 0.36]	0.10 [−0.15, 0.33]	−0.02 [−0.33, 0.29]	−0.07 [−0.37, 0.25]
2-back	0.09 [−0.15, 0.32]	0.08 [−0.17, 0.31]	0.38^a^ [0.08, 0.62]	−0.07 [−0.37, 0.25]
3-back	−0.03 [−0.27, 0.21]	−0.00 [−0.24, 0.24]	−0.23 [−0.51, 0.09]	0.07 [−0.24, 0.38]
IDP^b^				
Level 1	0.12 [−0.13, 0.35]	0.05 [−0.19, 0.29]	0.16 [−0.17, 0.45]	0.05 [−0.27, 0.36]
Level 2	0.06 [−0.18, 0.30]	0.00 [−0.24, 0.24]	0.09 [−0.23, 0.40]	0.12 [−0.20, 0.42]
Level 3	0.02 [−0.22, 0.26]	−0.11 [−0.34, 0.13]	0.20 [−0.12, 0.49]	0.22 [−0.10, 0.50]
Level 4	0.09 [−0.16, 0.32]	0.01 [−0.23, 0.25]	0.14 [−0.19, 0.43]	0.10 [−0.22, 0.40]
Level 5	−0.03 [−0.26, 22]	0.00 [−0.24, 0.24]	−0.14 [−0.44, 0.18]	0.22 [−0.11, 0.50]
Traditional measures				
SDMT	−0.08 [−0.31, 0.16]	−0.08 [−0.31, 0.16]	0.12 [−0.20, 0.42]	−0.06 [−0.37, 0.26]
Digit Span	−0.01 [−0.25, 0.22]	−0.21 [−0.42, 0.03]	0.00 [−0.31, 0.31]	0.12 [−0.20, 0.42]
RAVLT	−0.08 [−0.31, 0.16]	−0.06 [−0.30, 0.18]	0.06 [−0.25, 0.37]	0.11 [−0.21, 0.40]
TMT-Part B	0.11 [−0.13, 0.34]	0.15 [−0.09, 0.38]	−0.11 [−0.41, 0.21]	−0.10 [−0.40, 0.22]
COWAT	0.02 [−0.22, 0.26]	−0.01 [−0.25, 0.23]	−0.07 [−0.37, 0.25]	0.05 [−0.26, 0.36]

*Note.* Values in brackets represent the 95% confidence intervals around the estimate.

CCAMCHI = Cognitive Complaint After Mild Closed Head Injury; COWAT = Controlled Oral Word Association Test; IDP = Increasing Distractors Paradigm; mTBI = mild traumatic brain injury; RAVLT = Rey Auditory Verbal Learning Test; RPQ = Rivermead Postconcussion Symptoms Questionnaire; SDMT = Symbol Digits Modalities Test; TC = trauma control; TMT-Part B = Trail Making Test-Part B.

^a^This correlation was initially statistically significant (*p* < .05) but became non-significant after controlling for multiple comparisons.

^b^All correlations involving the IDP are partial correlations with baseline IDP performance controlled for.

In the mTBI group, correlations between cognitive symptoms and cognitive performance on the n-back and IDP ranged from |*r*| = 0.02–0.20, indicating that performance across levels explained an estimated 0.04%–4% of the variance in cognitive symptom reporting. In the TC group, performance across n-back and IDP levels explained an estimated 0%–14.44% of the variance in cognitive symptom reporting. These represent negligible to small effect sizes in the mTBI group, and negligible to medium effect sizes in the TC group. The average difference in correlation magnitude between mTBI and TC groups across task levels was 0.14, representing a small effect of higher correlations in the TC group.

Associations between cognitive performance and cognitive symptom reporting in mTBI were not statistically significant for measures of increasing cognitive load, or for traditional cognitive performance measures. The same pattern of null results was observed in the TC group after controlling for multiple comparisons. Correlations between cognitive performance and general post-concussion symptoms were also not significant for either group.

Given the potential confounding effects of psychological distress on the relationship between objective and subjective cognition (Burmester et al., 2016; Hromas et al., 2021), it is possible that psychological distress might have obscured a genuine relationship between these variables in bivariate analyses. For this reason, partial correlations were also conducted between cognitive performance and cognitive symptoms, controlling for the effects of psychological distress. To minimize the number of comparisons, these analyses were only conducted for the primary symptom measure of interest, the CCAMCHI. This data is presented in Table 4.

**Table 4:** Partial correlations between cognitive performance and cognitive symptoms, controlling for the effects of psychological distress

	mTBI (n = 67^a^)	TC (n = 38^a^)
n-back		
0-back	0.10 [−0.15, 0.33]	0.01 [−0.32, 0.33]
1-back	0.09 [−0.16, 0.32]	−0.02 [−0.34, 0.31]
2-back	0.09 [−0.15, 0.33]	0.39^b^ [0.07, 0.63]
3-back	−0.04 [−0.28, 0.20]	−0.25 [−0.53, 0.08]
IDP^c^		
Level 1	0.09 [−0.16, 0.33]	0.16 [−0.19, 0.46]
Level 2	0.09 [−0.16, 0.32]	0.10 [−0.24, 0.41]
Level 3	0.15 [−0.10, 0.38]	0.20 [−0.13, 0.50]
Level 4	0.17 [−0.08, 0.40]	0.14 [−0.20, 0.45]
Level 5	0.04 [−0.21, 0.28]	−0.14 [−0.45, 0.19]
Traditional measures		
SDMT	−0.07 [−0.30, 0.18]	0.12 [−0.21, 0.43]
Digit Span	0.02 [−0.22, 0.26]	0.00 [−0.32, 0.32]
RAVLT	−0.11 [−0.35, 0.13]	0.06 [−0.27, 0.38]
TMT-Part B	0.00 [−0.24, 0.24]	−0.11 [−0.42, 0.22]
COWAT	0.04 [−0.21, 0.27]	−0.07 [−0.39, 0.26]

*Note.* Values in brackets represent the 95% confidence intervals around the estimate.

CCAMCHI = Cognitive Complaint After Mild Closed Head Injury; COWAT = Controlled Oral Word Association Test; IDP = Increasing Distractors Paradigm; mTBI = mild traumatic brain injury; RAVLT = Rey Auditory Verbal Learning Test; RPQ = Rivermead Postconcussion Symptoms Questionnaire; SDMT = Symbol Digits Modalities Test; TC = trauma control; TMT-Part B = Trail Making Test-Part B.

^a^Following the exclusion one mTBI participant and two TC participants with missing psychological distress data.

^b^This correlation was initially statistically significant (*p* < .05) but became non-significant after controlling for multiple comparisons.

^c^All partial correlations involving the IDP control for baseline IDP performance in addition to psychological distress.

When controlling for psychological distress, performance across levels on the n-back and IDP explained an estimated 0.16%–2.89% of the variance in cognitive symptom reporting in the mTBI group (negligible to small effect sizes), and an estimated 0.01%–15.21% in the TC group (negligible to medium effect sizes).

There were no statistically significant associations between cognitive performance and cognitive symptoms in mTBI when controlling for the effects of psychological distress, both when employing measures of increasing cognitive load and when using traditional cognitive performance measures. Results in the TC group largely mirrored results observed in the mTBI group, with no significant associations between objective and subjective cognition after controlling for multiple comparisons.

It was noted that adding psychological distress as a covariate appeared to have very minimal effect on the correlation coefficients in the TC group (see similarities between Table 3 and Table 4 values for TCs). Given this observation, an exploratory analysis was undertaken to statistically compare the effect of controlling for psychological distress in each group. Specifically, difference scores were computed by subtracting partial correlation values from corresponding bivariate correlation values, and the absolute values (i.e., magnitude) of the difference scores were statistically compared between groups. The difference between groups was significant, *t*(13.61) = 4.10, *p* = .001, suggesting that psychological distress had a greater impact on the relationship between objective and subjective cognition in the mTBI group than in the TC group.

## DISCUSSION

This study found no significant associations between cognitive symptom reporting and cognitive performance at approximately 8 weeks following mTBI. Effect sizes were negligible to small in the mTBI group, with cognitive performance on each task level explaining less than 5% of the variance in cognitive symptom reporting. These findings were obtained despite employing cognitive performance measures proposed to be more sensitive than those previously employed to explore this relationship. A similar pattern of null results was found in the TC group after controlling for multiple comparisons, although estimated effect sizes were marginally higher in this group. There were also no significant associations between objective and subjective cognition in either group when controlling for the effects of psychological distress. These findings suggest that it is primarily factors aside from objective cognitive performance that underlie subjective cognitive symptom reporting in the post-acute period of mTBI.

The lack of significant association between performance and symptom reporting in this study was contrary to expectations, given the employment of two computer-based tasks of increasing cognitive load to measure cognition. Measures of this kind have been proposed to be more sensitive to subtle differences in cognitive performance (Bigler, 2013), and have been found to detect subtle cognitive deficits in post-acute mTBI when traditional neuropsychological measures were unable to detect these differences (Ettenhofer et al., 2020; Ozen & Fernandes, 2012; Vanderploeg et al., 2005). Research using these measures has also shown subtle cognitive deficits in a subsample of individuals reporting persistent cognitive symptoms after mTBI (Sparadeo et al., 2018). This study was the first to use computer-based tasks of increasing cognitive load to directly examine the relationship between objective and subjective cognition. In the context of these measures, the findings of this study do not provide support for a meaningful relationship between objective and subjective cognition at a group level at approximately 8 weeks post-mTBI.

Much of the existing research in this area has been conducted during the chronic period of injury, typically around 6–12 months post-injury (Clarke et al., 2012; Ngwenya et al., 2018; Stulemeijer et al., 2007). Thus, findings from these studies cannot be meaningfully compared to those of the current study, as cognitive performance after mTBI is dependent on time since injury (Karr et al., 2014). One of the few studies conducted within a comparable subacute period found a small but significant relationship between cognitive symptoms and performance on a memory task —but not other cognitive tasks —at 3 months following mTBI (Stenberg et al., 2020). However, this analysis did not control for psychological factors, which were significantly correlated with cognitive symptom reporting in the sample (Stenberg et al., 2020). Consequently, it remains an open question whether the identified relationship was independent of psychological factors. Another study examining individuals in the subacute period found weak and sporadic associations between cognitive performance and executive function symptoms, but no predictive relationship when controlling for psychological factors (Stillman et al., 2020). Thus, the findings of the current study are broadly consistent with previous research conducted during the subacute period of mTBI. The findings of the current study suggest that there is a disconnection between objective and subjective cognition 8 weeks after injury. This may be due to the fact that objective cognitive performance appears to have largely normalized by this time, despite ongoing subjective symptoms in many individuals in the current sample.

Although the current findings are clear at a group level, it remains possible that a genuine relationship between subjective and objective cognition exists in some individuals after mTBI, which was not identified by the current study. Specifically, it is possible that a significant relationship may exist for a subset of individuals—those with poor recovery after mTBI—with effects obscured by group analyses (see Iverson, 2010). Another possibility is that subjective cognitive symptoms in the post-acute period represent, at least in part, true cognitive changes that cognitive performance measures are unable to detect. That is, individuals with cognitive symptoms after mTBI may be able to perform normally even on complex cognitive tasks, but find that it takes them more effort to do so and requires the use of compensatory strategies (Jessen et al., 2014). The results of this study cannot differentiate between these or other possibilities, and further research would be required for clarity in this area. However, given that a large body of existing research has not yet been able to answer these questions, novel approaches may be required.

Despite these possible explanations, the null statistical findings in this study, in the presence of cognitive performance measures proposed to be more sensitive than traditional tasks, most strongly suggest that at a group level it is factors aside from objective cognition that are primarily driving cognitive symptoms in post-acute mTBI. These conclusions are further supported by the negligible to small effect sizes seen in this study, showing that cognitive performance across levels explained only minimal amounts (<5%) of the variance in cognitive symptoms in the mTBI group. Given these findings, it is necessary for research in mTBI to continue to explore other factors that might contribute more significantly than objective cognition to persistent cognitive symptoms in the post-acute period of injury.

A key component of this study was the inclusion of a group of individuals with general trauma, in order to provide a better understanding of the relationship between objective and subjective cognition in mTBI. The results of the TC group generally mirrored the results of the mTBI group in this study, with no significant relationships found between objective and subjective cognition in the TC group after controlling for multiple comparisons. When considering effect sizes, estimated effects in the TC group appeared marginally larger on average than effects in the mTBI group, although these were not directly compared statistically.

The overall similar pattern of results between mTBI and TC groups in this study suggests that the lack of significant relationship between objective and subjective cognition in mTBI may not be unique to this population. Although not always consistent, previous research has found various relationships between subjective and objective cognition in healthy adults (Hess et al., 2020; Langlois & Belleville, 2014; Zlatar et al., 2022). If a robust relationship between objective and subjective cognition is present in healthy individuals, but not in mTBI or TC groups, this suggests that factors associated with general trauma may contribute to this lack of relationship in mTBI. General trauma is known to affect both symptom reporting and performance (Cassidy et al., 2014; McCauley et al., 2014). Thus, it is possible that general trauma may also affect the *relationship* between the two factors. Further research with injured and healthy controls is warranted to understand whether the experience of mTBI and/or general trauma result in a change to the “normal” relationship between objective and subjective cognition.

When controlling for the potential confounding effects of psychological distress on the relationship between objective and subjective cognition, there were no changes to the overall pattern of results. This provides further corroboration for a lack of meaningful independent relationship between cognitive performance and cognitive symptom reporting at a group level 8 weeks after mTBI.

Further, exploratory t-tests showed that covarying for psychological distress had a significantly a greater impact on correlation values in the mTBI group than in the TC group. This suggests that psychological distress may play a greater role in the relationship between objective and subjective cognition in mTBI groups than in TCs.

These findings are clinically relevant as they emphasize the complexity of subjective cognitive symptoms, and suggest that individuals presenting with these symptoms in the post-acute period of mTBI may not necessarily have detectable cognitive impairments. Rather, other factors not related to cognitive performance may be driving cognitive symptoms in many individuals who present clinically with these subjective difficulties.

In the current samples, group scores on each psychological distress measure fell below recommended thresholds for clinical anxiety, depression, and PTSD. The recruitment of premorbidly healthy adults in the current study may have mitigated against post-injury psychological distress, given the strong relationship between premorbid and post-injury psychological issues (Yue et al., 2019). Nevertheless, the current subthreshold psychological distress levels are not out of keeping with previous research: although psychiatric problems are common after mTBI (Carroll et al., 2014), clinical elevation is not typically observed at a group level (de Koning et al., 2016; Ponsford et al., 2011). This pattern does not preclude the possibility of clinical levels of psychological distress post-injury in some individuals within the current sample, however.

These results are likely to generalize to other samples of premorbidly healthy civilians who were admitted to hospital after mTBI. Although premorbid health issues are common in those who experience mTBI (Yue et al., 2019; Zeldovich et al., 2020), the recruitment of premorbidly healthy adults in the current study allowed for the examination of mTBI in an isolated manner, removing the impact of premorbid health issues as a potential confounder. This does however impact the generalizability of the current results, with findings not necessarily generalizing to those with premorbid health issues. Given that this sample was recruited from inpatient trauma wards, findings also may not generalize to the many individuals with mTBI who do not present to hospital or who do not require hospital admission (Cassidy et al., 2004; Gardner & Yaffe, 2015). Importantly, individuals in the current samples were admitted to hospital due to general traumatic injuries and not due to their mTBI, so did not by definition have more severe *brain injuries* than other samples. Nevertheless, given the nature of the current sample, findings are most likely to generalize to individuals with mTBI who were premorbidly healthy and suffered other bodily injuries requiring admission.

The primary limitation of this study is the sample size, which was modest in each group, although similar to previously published studies in the area (Cassidy et al., 2014). Sample size contributes to the statistical power of studies, and smaller samples can lead to reduced statistical power, which can obscure genuine relationships (Field, 2012). In this study, the reporting and interpretation of effect sizes alongside tests of statistical significance served to buffer the potential consequences of the modest sample size, given that effect sizes are themselves independent from sample size (Sullivan & Feinn, 2012). The low estimated effect sizes suggest that the null statistical findings of the current study are not simply a result of small sample size.

Additionally, although the mTBI and TC groups were overall very well-matched, psychological distress was significantly higher in the mTBI group when a TC outlier was removed. Given the importance of psychological factors in the relationship between objective and subjective cognition (Burmester et al., 2016; Hromas et al., 2021), the difference in psychological distress between groups may have affected the pattern of bivariate correlation results between the mTBI and TC groups. Nevertheless, when psychological distress was controlled for using partial correlations, the overall pattern of results between groups was unchanged. This provides support for the robustness of this pattern irrespective of the potential impact of psychological distress.

Finally, summary cognitive performance data for the 0-back and 3-back levels of the n-back suggests a possible ceiling effect and a floor effect of performance, respectively, with low variance on both levels. A ceiling effect of performance on the 0-back has been observed previously in an mTBI sample (Dean & Sterr, 2013). Given that variance is crucial for establishing a relationship between two variables, it is possible that correlations on these specific levels were attenuated by the restriction of range (Goodwin & Leech, 2006; Schmiedek et al., 2014). There was no evidence for a restriction of range on the remaining n-back levels, or on any level of the IDP, thus this cannot account for the low correlations on these other levels.

## CONCLUSIONS

This study was the first to employ computer-based tasks of increasing cognitive load (proposed to be more sensitive than traditional measures) to directly examine the relationship between objective and subjective cognition in post-acute mTBI. No significant associations were found at a group level between cognitive performance and cognitive symptoms in mTBI at approximately 8 weeks post-injury, suggesting that it is primarily factors aside from objective cognition that underlie elevated cognitive symptom reporting in the post-acute period of mTBI. This pattern of null results continued to be seen when controlling for psychological distress, a key confounding variable in the relationship between objective and subjective cognition. The pattern of results in the TC group generally mirrored the results of those observed in the mTBI group, suggesting that this disconnect between objective and subjective cognition may not be specific to mTBI. Exploratory analyses showed that psychological distress played a larger role in the relationship between objective and subjective cognition in the mTBI group than in the TC group, suggesting that the factors affecting this relationship may show a degree of specificity. To better support individuals with poor recovery after mTBI, future research should continue to explore novel factors aside from objective cognition that may be underlying ongoing cognitive symptom reporting in this group.
